# Overexpression of TRIM24 Is Associated with the Onset and Progress of Human Hepatocellular Carcinoma

**DOI:** 10.1371/journal.pone.0085462

**Published:** 2014-01-07

**Authors:** Xiao Liu, Yu Huang, Dinghua Yang, Xianghong Li, Jiankun Liang, Liang Lin, Meng Zhang, Kebo Zhong, Bo Liang, Jialu Li

**Affiliations:** Department of Hepatobiliary Surgery, Nanfang Hospital, Southern Medical University, Guangzhou, China; Hong Kong University of Science and Technology, China

## Abstract

The survival and colonization of tumor cells at new locations involve a variety of complex genetic, epigenetic, and microenvironmental factors. TRIM24 was originally named transcription intermediary factor 1-alpha (TIF1α), which was associated with cellular proliferation and was an oncogene in tumor development. Here we provide the first evidence of the expression profile and clinicopathological significance of TRIM24 in patients with hepatocellular carcinoma (HCC). Immunohistochemistry was employed to determine the expression level of TRIM24 in HCC tissues and noncancerous liver tissues. Elevated TRIM24 level was found in 61.4% HCC samples (51/83) correlating with AFP (P = 0.036), poor differentiation (P = 0.004), intrahepatic metastasis (P = 0.004), recurrence (P = 0.000006), and shorter tumor-free survival time (P = 0.002). Small interfering RNA induced down-regulation of TRIM24 promoted apoptosis in HCC cell line HepG2. Moreover, western blotting analysis revealed that knockdown of TRIM24 increased the protein levels of p53, Bax, and Caspase-8, and decreased Bcl-2, Survivin, Cyclin D1, and CDK4. Depletion of TRIM24 decreased Snail, Slug, β-catenin, and Vimentin, and increased E-cadherin expression, which suggested the involvement of TRIM24 in EMT. These results indicated that TRIM24 plays an important role in the pathogenesis of human HCC.

## Introduction

Hepatocellular carcinoma (HCC) is one of the most common malignancies, affecting over one million people annually worldwide with a mortality rate almost equal to its incidence [1], [2]. In spite of recent advances in diagnosis and treatment, the long-term outcome for HCC patients after effective treatment still remains generally poor, for which the high rate of tumor recurrence and lack of effective therapeutic strategy for patients with recurrent HCC are blamed [3], [4]. One of the main reasons for the high early recurrence after curative hepatectomy in HCC patients is frequent intrahepatic metastasis [5]. It would be beneficial clinically to discover biological markers that predict early tumor relapse, identify their molecular mechanisms in prioritising HCC patients with high potential for tumor recurrence, and then design a better therapeutic target.

TRIM24, formerly known as transcription intermediary factor 1-alpha (TIF1α), a member of the tripartite motif protein family, was among the first co-regulators identified as interacting with RARs in ligand-dependent fashion [6]–[8]. Existing research showed that down-regulation of TRIM24 might promote tumor development through complex mechanisms. More recently, TRIM24 has been identified as a novel partner of the p53 tumor suppressor which negatively regulates p53 protein [9], [10]. Indeed, TRIM24 directly ubiquitinates p53 via its RING domain, and depletion of TRIM24 in human breast cancer cells induced p53-dependent apoptosis. TRIM24 could also bind chromatin and oestrogen receptor to activate oestrogen-dependent genes so as to regulate cellular proliferation and tumor progress [11], [12]. In acute promyelocytic leukaemia, myeloproliferative syndrome and papillary thyroid carcinoma, TRIM24 plays an important role as a target of chromosomal translocations to form oncogenic fusion proteins [13]–[15]. It has been reported that overexpression of TRIM24 could promote prostate cancer progress, and high level of TRIM24 proteins was associated with poor prognosis in breast cancer [16]. Moreover, overexpression of TRIM24 was correlated with pTNM stage and differentiation in non-small cell lung cancer [17]. These finding suggested that TRIM24 may promote tumor progress. On the other hand, recent studies showed that loss of TRIM24 in mice induces tumor development and that TRIM24 interacted with TRIM28 and TRIM33 to form regulatory complexes that suppressed murine hepatocellular carcinoma [18]. The protein expression of TRIM24 in human primary hepatocellular carcinoma and its relationship with clinicopathological factors have not yet been reported and, therefore, need to be discovered. In addition, the biological roles of TRIM24 in hepatocellular carcinoma are still unclear. In this study, we report the TRIM24 expression in human hepatocellular carcinoma tissues by immunohistochemistry and effects of TRIM24 in apoptosis, cell cycle, and EMT in hepatocellular carcinoma cell line.

## Results

### Increased Expression of TRIM24 Protein in Human Hepatocellular Cancer Tissues

**Figure 1:**
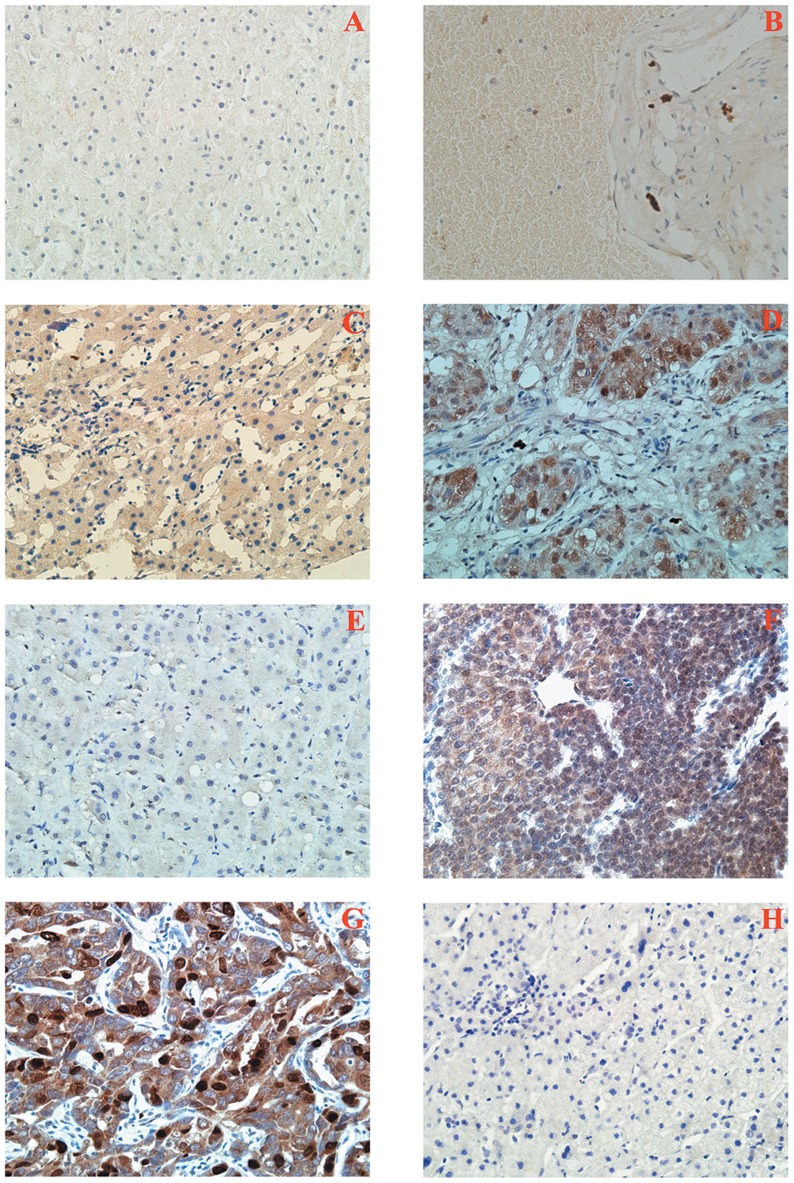
Immunohistochemical staining of TRIM24 in tissue sections. A. Negative staining in normal liver tissue. B. Negative staining in benign liver lesions tissues (Hepatic hemangioma). C. Negative TRIM24 staining in an AFP>400 ug/L, well differentiated HCC tissue. D. Positive TRIM24 staining in an AFP<400 ug/L, moderate differentiated HCC tissue. E. Negative TRIM24 staining in an AFP<400 ug/L,well differentiated HCC tissue. F&G. Positive TRIM24 staining in an AFP>400 ug/L, poor differentiated HCC tissue. H. Negative control using antibody diluent.

**Figure 2:**
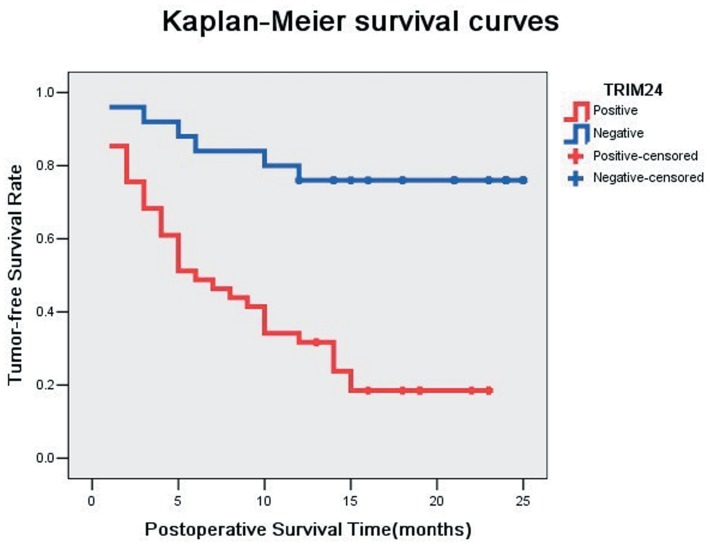
The Kaplan-Meier survival curves of patients with positive TRIM24 expression and negative TRIM24 expression.

We analyzed the expression of TRIM24 protein in 83 HCC specimens and their corresponding normal tissues by immunohistochemistry. TRIM24 expression was detected in nuclear compartments of tumor cells (Figure 1 D, F–G), while normal tissues or benign liver lesion tissues exhibited negative or low staining (Figure 1 A–B). The staining intensity of liver tissues adjacent to a tumor could be evaluated in several sections containing malignant tumors and normal tissues in the same slide. A strong staining of TRIM24 was detected in adjacent tumor cells, while negative or low staining of TRIM24 was detected in the normal liver tissues. The relationship between the total TRIM24 expression and the clinicopathological factors is shown in Table 1: no statistical difference was found between the TRIM24 overexpression and the characteristics of TNM stage (P = 0.108), BCLC stage (P = 0.535), gender (P = 0.614), Age (P = 0.066), Hepatitis B (P = 0.416), Tumor size (P = 0.967), Tumor amount (P = 0.277), Lymph node metastasis (P = 0.553), Tumor capsule (P = 0.337), Tumor thrombus (P = 0.103), and Cirrhosis (P = 0.065). More interestingly, patients with TRIM24 overexpression showed poor differentiation (P = 0.004), higher level of AFP (P = 0.036), higher incidence of intrahepatic metastasis (P = 0.004) and recurrence (P = 0.000006), and shorter Tumor-free survival time (P = 0.002) (Figure 2).

**Table 1:** Relationship between the TRIM24 expression and the clinicopathological factors.

Characteristics	Number of patients	TRIM24 negative	TRIM24 positive	*χ^2^*	*P*
TNM stage				2.580	**0.108**
I+II	62	27(43.5%)	36(56.5%)		
III+IV	21	7(23.8%)	16(76.2%)		
BCLC stage				1.253	**0.535**
A	51	21(41.2%)	30(58.8%)		
B	11	5(45.5%)	6(54.5%)		
C	21	6(28.6%)	15(71.4%)		
Gender				0.255	**0.614**
Male	72	27(37.5%)	45(62.5%)		
Female	11	5(45.5%)	6(54.5%)		
Age				3.367	**0.066**
<60	72	25(34.7%)	47(65.3%)		
≥60	11	7(63.6%)	4(36.4%)		
Hepatitis B^a^				0.662	**0.416**
Negative	8	2(25.0%)	6(75.0%)		
Positive	73	29(39.7%)	44(60.3%)		
AFP (ug/L)^a^				4.416	**0.036**
<400	54	25(46.3%)	29(53.7%)		
≥400	27	6(22.2%)	21(77.8%)		
Tumor size (cm)				0.068	**0.967**
<3.0	15	6(40.0%)	9(60.0%)		
3.0≤X<5.0	25	10(40.0%)	15(60.0%)		
≥5.0	43	16(37.2%)	27(62.8%)		
Amount				1.183	**0.277**
1	62	26(41.9%)	36(58.1%)		
≥1	21	6(28.6%)	15(71.4%)		
Lymph node metastasis				0.351	**0.553**
No	73	29(39.7%)	44(60.3%)		
Yes	10	3(30.0%)	7(70.0%)		
Tumor capsule^b^				0.924	**0.337**
No	14	4(28.6%)	10(71.4%)		
Yes	66	28(42.4%)	38(57.6%)		
Tumor thrombus				2.661	**0.103**
No	68	29(42.6%)	39(57.4%)		
Yes	15	3(20.0%)	12(80.0%)		
Cirrhosis^c^				3.412	**0.065**
No	25	6(24.0%)	19(76.0%)		
Yes	57	26(45.6%)	31(54.4%)		
Differentiation				10.949	**0.004**
Well	19	12(63.2%)	7(36.8%)		
Moderate	38	16(42.1%)	22(57.9%)		
Poor	26	4(15.4%)	22(84.6%)		
Intrahepatic metastasis^d*^				8.187	**0.004**
No	41	21(51.2%)	20(48.8%)		
Yes	25	4(15.4%)	21(84.6%)		
Recurrence^d^				20.500	**0.000006**
No	27	19(70.4%)	8(29.6%)		
Yes	39	6(15.4%)	33(84.6%)		
Recurrence time (months)^d^				12.307	**0.002**
<6	25	4(16.0%)	21(84.0%)		
6≤X<12	9	2(22.2%)	7(77.8%)		
≥12	32	19(59.4%)	13(40.6%)		

Table 1: a. Missing data for 2 patients. b. Missing data for 3 patients. c. Missing data for 1 patient. d. Missing data for 10 patients; 7 patients were excluded according to the inclusion criteria. *.We defined that those patients whose recurrence time is less than 6 months as intrahepatic metastasis.

### Depletion of TRIM24 Induces Apoptosis in HepG2

Since the role of TRIM24 is closely associated with p53 in some types of tumor, as reported above, we examined p53 expression in HepG2, a hepatocellular cell line which expresses wild type p53. In order to explore the biological function of TRIM24 in HCC, 2 siRNAs (siTRIM24(1) and siTRIM24(2)) of different sequences were employed to knockdown TRIM24 expression in HepG2 cell line. TRIM24-specific siRNA considerably reduced protein expression levels of TRIM24 after 48 hours of siRNA treatment.

**Figure 3:**
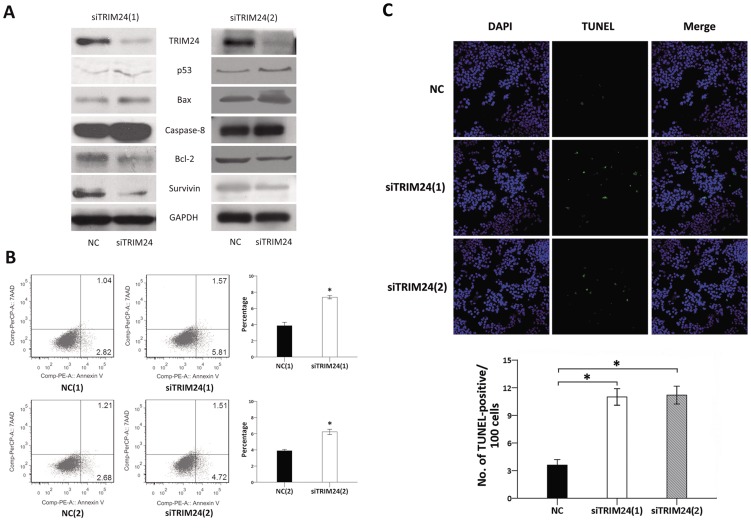
TRIM24 depletion induces apoptosis in HepG2. 3(A). Western blotting analysis of the cell-apoptosis related proteins showed the expression of p53, Bax, and Caspase-8 were increased, and Bcl-2 and Survivin were decreased after knockdown TRIM24 in HepG2 cells; 3(B) TRIM24 knockdown induced apoptosis in HepG2 cell line(P<0.05); 3(C) TUNEL analysis of cell apoptosis showed that TRIM24 silencing increased apoptosis in HepG2.

We detected the apoptosis-related proteins by western blotting. The results revealed that knockdown of TRIM24 increased the protein levels of p53, Bax, and Caspase-8, and decreased Bcl-2 and Survivin (Figure 3A). In addition, Annexin V kit was employed to characterize the death feature of HepG2 cells with TRIM24 knockdown. Clearly, a significant population of early and late apoptosis (5.81% to 2.82%, and 1.57% to 1.04%, all P<0.05) were observed in cells with TRIM24(1) knockdown by siTRIM24 compared with scramble control (Figure 3B). Similarly, there was a significant population of early and late apoptosis (4.72% to 2.68%, and 1.51% to 1.21%, all P<0.05) in cells treated with siTRIM24(2) compared to scramble control (Figure 3B). In the TUNEL assay, cell apoptosis was increased in treated group compared with scramble group (P<0.05) (Figure 3C); this demonstrated that TRIM24 knockdown induced apoptosis of p53 dependent in hepatocellular cells.

### Depletion of TRIM24 Blocks Cell Cycle in HepG2

**Figure 4:**
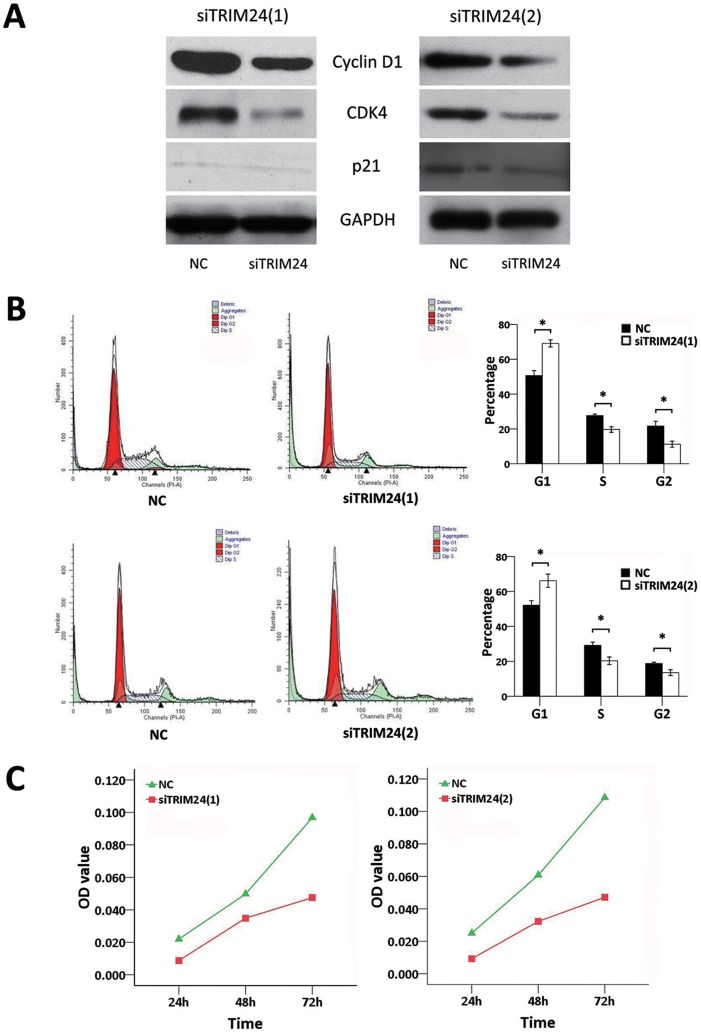
Depletion of TRIM24 reduces cell proliferation. 4(A). Western blotting analysis of the cell-cycle related proteins showed the expression of Cyclin D1 and CDK4 were decreased but showed no significant change in p21 after knockdown TRIM24 in HepG2 cells; 4(B). Cell cycle analyses showed that the percentage of G1 phase was increased in siTRIM24 group (P<0.05), whereas the percentages of S phase (P<0.05) and G2 phase (P<0.05) were decreased in the TRIM24 knockdown cells compared with control cells; 4(C) CCK-8 assay suggested that cell proliferation of HepG2 after TRIM24 silencing was reduced compared with the control group.

The cell cycle related proteins were analyzed with and without TRIM24 knockdown, and it was found that depletion of TRIM24 down-regulated Cyclin D1 and CDK4, but did not affect p21 (Figure 4A). Further, the cell cycle analyses were performed in HepG2 with or without siTRIM24 transfect. We found that the percentage of G1 phase (69.13% compared with 50.67% in siTRIM24(1) group, P<0.05; 66.15% compared with 52.11% in siTRIM24(2) group, P<0.05) increased in cells dealt with siTRIM24, whereas the percentage of S phase (27.66% compared with 19.67% in siTRIM24(1) group, P<0.05; 29.15% compared with 20.33% in siTRIM24(2) group, P<0.05) decreased in the siTRIM24 group compared with the negative control group (Figure 4B). Also, the percentage of G2 phase cells decreased (21.67% compared with 11.20% in siTRIM24(1) group, P<0.05; 18.73% compared with 13.52% in siTRIM24(2) group, P<0.05). These results indicated that the cell cycle may be blocked at the G1/S boundary by down-regulation of TRIM24. Moreover, the CCK-8 assay was employed to investigate cell proliferation in HepG2 cells. After being transfected with siTRIM24, cell proliferation markedly decreased in HepG2 cells (Figure 4C).

### Depletion of TRIM24 Inhibits the Process of EMT

**Figure 5:**
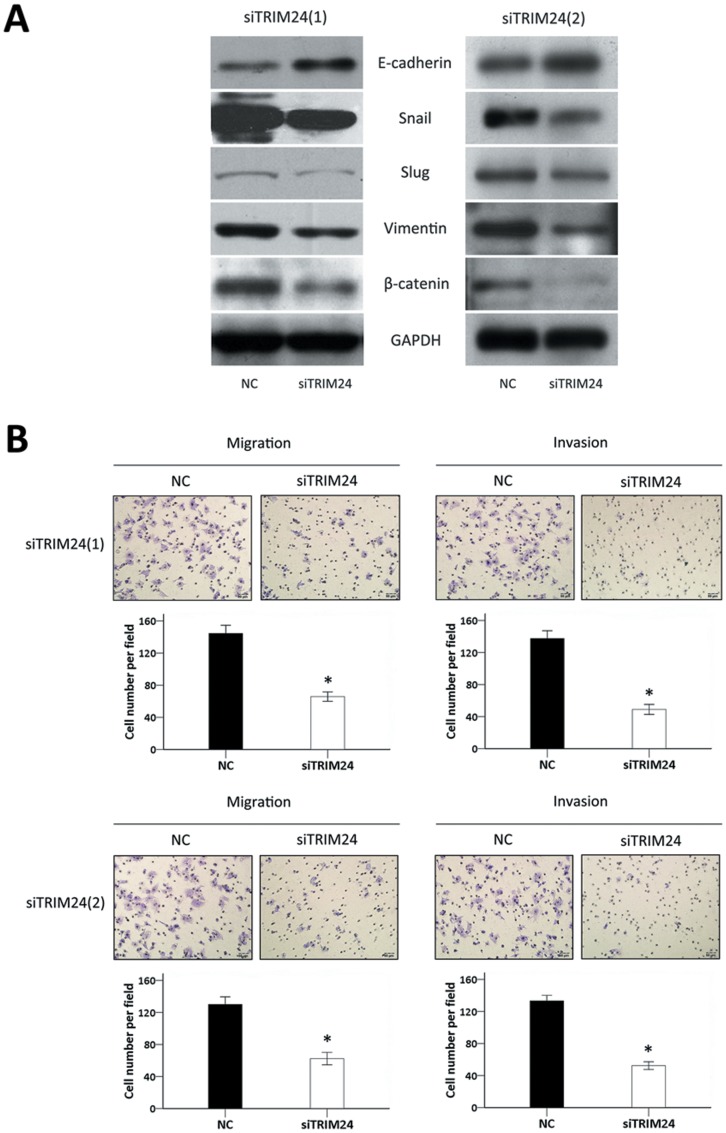
Depletion of TRIM24 inhibits the process of EMT. 5(A). Western blotting analysis of the cell-apoptosis related proteins showed the expression of E-cadherin was increased and Snail, Slug, Vimentin, andβ-catenin were decreased after knockdown TRIM24 in HepG2 cells; 5(B). Reduced migration and invasion ability of HepG2 cells at 48 h post-transfection with siTRIM24 (P<0.05, compared with controls).

The expression of EMT-related proteins such as E-cadherin, β-catenin, Snail, Slug, and Vimentin, was analyzed by Western blot. After knockdown of TRIM24, the expressions of E-cadherin was up-regulated, while β-catenin, Snail, Slug, and Vimentin were down-regulated in each siTRIM24 group compared with the negative control group (Figure 5A). Transwell migration assay was performed to measure migration and invasion ability. It was found that the migration and invasion ability of HepG2 cells was reduced at 48 h after being transfected with siTRIM24 (P<0.05, compared with controls) (Figure 5B). These results suggested that TRIM24 may play an important role in EMT in human hepatocellular carcinoma.

## Discussion

Recent research in acute promyelocytic leukaemia, myeloproliferative syndrome, papillary thyroid carcinoma, breast cancer, and non-small cell lung cancer has shown that the expression of TRIM24 was up-regulated [13]–[17]. In addition, overexpression of TRIM24 is correlated with survival of breast cancer patients and with pTNM stage and differentiation in non-small cell lung cancer. These researches suggested that the TRIM24 gene might play an important role in tumorigenesis because it encodes a protein that controls nuclear receptor ligand-dependent activity [19] and acts as a ubiquitin E3-ligase for the tumor suppressor p53 [9].

TRIM24 suppressed tumorigenesis during retinoic acid receptor activation to prevent liver cancer in the murine model [20], [21]. However, the protein of TRIM24 expression in human HCC, and the relationship between TRIM24 expression in human HCC and clinicopathological factors, has not yet been defined. In our study, we demonstrated that the protein expression of TRIM24 in HCC cancer tissues was higher than in noncancerous tissues, normal tissues, and benign liver lesions tissues. There was a close correlation between TRIM24 up-regulation and differentiation (P = 0.004), AFP (P = 0.036), intrahepatic metastasis (P = 0.004), and recurrence (P = 0.000006). Patients with TRIM24 overexpression showed poor differentiation, higher level of AFP, higher incidence of intrahepatic metastasis and recurrence, and shorter Tumor-free survival time. These findings suggested that TRIM24 may play an important role in human HCC progress.

Previous studies indicated that TRIM24 could directly ubiquitinate p53 and negatively regulate protein level of p53 in breast cell lines, implying its roles in proliferation and apoptosis [9]. In order to confirm the potential role of TRIM24 in human HCC development, we employed siRNA to knockdown TRIM24 in HepG2 cell line which express wild type p53 for further study. First, we detected the expression of apoptosis-related proteins such as p53, Bcl-2, Bax, Caspase-8, and Survivin by western blotting and found that depletion of TRIM24 up-regulated p53, Bax, and Caspase-8, and down-regulated Bcl-2 and Survivin. We also characterized the death feature of HepG2 cells with TRIM24 knockdown using Annexin V kit. Our results showed a significant increase in population of apoptosis (7.38% compared with 3.86% in siTRIM24(1) group, P<0.05; 6.23% compared with 3.89% in siTRIM24(2) group, P<0.05) in cells with TRIM24 knockdown as compared to negative controls from the cell apoptosis by flow cytometry. The same tendency was observed in western blotting detection and TUNEL analysis of cell apoptosis. These results suggested that TRIM24 may suppress cell apoptosis and differentiation via ubiquitinating p53. Wild type p53 also plays an important role in cell cycle, differentiation, and apotosis [22]. The above results might explain the mechanism of TRIM24 on human HCC cell differentiation.

Since most of the proliferative factors could influence cell growth by affecting cell cycle progress, we employed cell cycle analysis and CCK-8 assay to investigate the role TRIM24 performed in cell proliferation. The results showed a higher percentage of G1 phase and lower S phase and G2 phase in the siTRIM24 groups than the control groups. Cell proliferation of HepG2 after TRIM24 silencing was down-regulated compared with the control group, which suggested that TRIM24 might promote cell proliferation in HepG2 cells. Western blotting was employed to detect the expressions of cell-cycle related proteins including Cyclin D1, CDK4, and p21. Complex phosphorylating Rb, a key factor that regulates cell proliferation by controlling the restriction point within G1 phase, is also produced by Cyclin D1 and CDK4 [23]. Overexpression of Cyclin D1 was observed in numerous cancers and proved to be associated with cancer cell proliferation [24]–[26]. We found a remarkable reduction of Cyclin D1 and CDK4 level, which was correlated with the increased percentage of G1 phase and decreased S and G2 phase. Thus, we suggested that TRIM24 might be a pivotal mediator in the cell cycle control of HCC cells.

Epithelial to mesenchymal transition (EMT) under stimulation is correlated with tumor invasion and metastasis. During EMT, epithelial cells lose polarity and gain more ability of migration [27]. We detected the expression of EMT regulatory molecules such as E-cadherin, Snail, Slug, Vimentin, andβ-catenin in HCC using western blot, and measured the migration and invasion ability of HepG2 cells in both the siTRIM24 groups and the negative control groups using Transwell assay. The results showed that depletion of TRIM24 up-regulated the protein levels of E-cadherin and down-regulated Snail, Slug, Vimentin, and β-catenin. That meant the EMT has been blocked after knockdown TRIM24; and the migration and invasion ability was obviously decreased after TRIM24 silencing. It was suggested that TRIM24 could play an important role in the EMT progress. Moreover, this could be the explanation for the result that positive of TRIM24 is correlated with higher incidence of intrahepatic metastasis and recurrence in immunohistochemistry.

In conclusion, this study investigated the expression and clinopathological significance of TRIM24 in human hepatocellular carcinoma. Further work is warranted to elucidate the mechanism of TRIM24 acting in the pathogenesis of hepatocellular carcinoma.

## Materials and Methods

### Patients and Tissues

83 cases of HCC samples were obtained from the Hepatobiliary Surgery of Nanfang Hospital of Southern Medical University during the period 2009–2012. The histological diagnosis and grade of differentiation of the tumors were evaluated by the department of pathology of Nanfang Hospital, according to the World Health Organization standard. The study was performed in accordance with the ethical standards of the Declaration of Helsinki and was approved by the Ethical Committee of Nanfang Hospital in Southern Medical University. All subjects gave written, informed consent.

### Immunohistochemistry

HCC tissue sections were embedded in paraffin and 4 mm-thick sections were prepared after being fixed with 10% neutral formalin for more than 24 hours. Two-step immunohistochemical method (PV-9000, ZSGB-BIO, China) was employed to perform the immunostaining. After being deparaffinized in xylene and rehydrated in graded alcohol series (100%, 95%, 85%, 75%) for two minutes each, the sections were boiled in citrate buffer for 10 minutes in a pressure-cooker. Hydrogen peroxide (0.3%) was used to block the endogenous peroxidase activity of the tissue sections. Then PBS was used to wash the sections three times, three minutes each. Tissue sections were incubated with TRIM24 mouse monoclonal antibody (1∶100) (Abnova, Taiwan). 0.5% BSA was used as a negative control. Staining for the TRIM24 primary antibodies was performed at 4°C for the whole night. Biotinylated goat anti-mouse serum IgG (ZSGB-BIO, PV-9000, China) was used as the secondary antibody. After washing, the sections were incubated with horseradish peroxidase-conjugated streptavidin–biotin, followed by 3, 3′-diaminobenzidine tetrahydrochloride to develop the peroxidase reaction. Counterstaining of the sections was done with hematoxylin, before they were dehydrated in graded ethanol prior to mounting. Hematoxylin was used to counterstain the sections. The sections were then dehydrated in graded ethanol (75% for two minutes, 85% for two minutes, 95% for two minutes, 100% for two minutes, 100% for five minutes). Neutral resin was used to seal the coverslips afterwards.

Two independent investigators examined all tumor slides randomly. Five views were examined per slide, and 100 cells were observed per view at 400× magnification. Immunostaining of TRIM24 was scored following a semi-quantitative scale by evaluating, in representative tumor areas, the intensity and percentage of cells showing higher immunostaining than the control cells. Nuclear staining of the tumor cells was considered as positive immunostaining. The intensity of TRIM24 nuclear staining was also scored as 0 (no staining), 1 (weak), 2 (marked). Percentage scores were assigned as: 1, 1–25%; 2, 26–50%; 3, 51–75%; 4, 76–100%. The scores of each tumor sample were multiplied to give a final score of 0 to 8 and the total expression of TRIM24 was determined as either negative or low expression (−): score <4 or overexpression (+): score ≥4 [17].

### Cell Line and Culture

Human liver cancer cell line HepG2 (Shanghai Institutes for Biological Sciences of the Chinese Academy of Sciences, China) was maintained in monolayer cultures in high glucose Dulbecco’s Modified Eagle Medium (DMEM, Hyclone, Thermo Scientific, USA) containing 10% (v/v) foetal bovine serum (FBS, Hyclone, Thermo Scientific, USA) at 37°C in a humidified atmosphere of 5% CO_2_. Cells in the exponential growth phase were harvested and a cell suspension was prepared at a density of 3.0×10^7^ cells/mL and added into 6-well plates for transfection.

### Cell Transfection

HepG2 cells were assigned to siRNA-TRIM24 group, blank group, and control groups. Cells in siRNA-TRIM24 groups were transfected with two different sequences: siRNA- TRIM24 (siTRIM24(1) 5′-3′ GAGCUCAUCAGAGGGUAAATT, 5′-3′ UUUACCCUCUGAUGAGCUCTT; siTRIM24(2) 5′-3′ GCCACCAAGUGGUUUAUCATT, 5′-3′ UGAUAAACCACUUGGUGGCTT) (GenePharma, China) – according to manufacturer’s instructions. Cells in the blank group were exposed to normal medium. Cells in the control groups were exposed to either X-tremeGENE siRNA transfection reagent (Roche, Switzerland) or NC siRNA. All transfections were carried out in triplicate. siRNA- TRIM24 were mixed with X-tremeGENE siRNA transfection reagent (Roche, Switzerland) followed by a 20-minute incubation. The concentration of siRNA- TRIM24 was diluted to 50 nmol/L before transfection. Cells were incubated with 200 ul of transfection mixture and serum-free medium, and collected after 48 hours.

### Detection of Selected Genes Protein Expression

Total protein was extracted at 48 hours after the transfection with Total Protein Extraction Kit (BioChain, USA), according to manufacturer’s instructions. In brief, 100 ug of proteins in each group was subjected to 10% SDS/PAGE and transferred onto PVDF membranes (Roche, Switzerland). The membranes were subsequently incubated with antibodies: TRIM24 (1∶500) (Abnova, Taiwan); p53 (1∶500), Snail (1∶500) (Abcam, England); Cyclin D1 (1∶200), CDK4 (1∶200), Vimentin (1∶200), β-catenin (1∶200), E-cadherin (1∶200) (Santa Cruz, USA); Bax (1∶1000), Bcl-2 (1∶1000), Survivin (1∶1000), Slug (1∶1000) (Cell signaling technology, USA), Caspase-8 (1∶200), p21 (1∶200) (BD Pharmingen, USA), and GAPDH (1∶1000; Santa Cruz, USA) at 4°C for the whole night after incubation in 5% BSA for two hours. After the incubation with horseradish peroxidase conjugated secondary antibody (1∶2000–10000; Abcam, USA) for two hours at room temperature, visualization was performed with an enhanced chemiluminescence kit (Millipore, USA) followed by exposure to X-ray film (Kodak, USA). GAPDH served as an endogenous control. This experiment was performed three times.

### Cell Proliferation Assay

CCK-8 assay was conducted to measure cell proliferation. Cells were seeded in a 96-well plate at 0.5×10^4^/well for 24 hours, then transfected with TRIM24 siRNA or NC siRNA. The transfected cells were incubated for 24, 48 and 72 hours respectively. Ten microliters of CCK-8 reagent was added to each well two hours before the end of the incubation. Following the incubation, the absorbance of each well at 450 nm was determined using a microplate reader.

### Detection of Apoptosis and Cell Cycle by Flow Cytometry

Cell apoptosis was measured by FACS analysis using PE Annexin V Apoptosis Detection Kit, according to the manufacture’s instructions (BD Biosciences, CA, USA). Briefly, 1×10^6^ HepG2 cells were collected at 48 hours after the transfection with TRIM24 siRNA or NC siRNA by centrifugation at 300×g for five minutes at room temperature. Cells were washed once in cold PBS, gently re-suspended in 100 µl 1× binding buffer and incubated with Annexin-V-PE and 7AAD in dark for 15 minutes at room temperature. Then 400 µl 1× binding buffer was added to the cell suspension and fluorescence-activated cell sorting analysis was carried out using a FACS Canto II flow cytometer and Cell Quest software.

Similarly, TRIM24’s effect on HepG2 cell cycle was also analyzed via FACS approach. Transfected cells were washed in cold PBS and re-suspended into 1 ml of 70% ethanol, and then fixed overnight. Then the ethanol-suspended cells were collected, washed, and stained with 50 µg/ml propidium iodide containing 250 µg RNase A in the dark for 30 minutes at room temperature, and subsequently analyzed using a FACS Canto II flow cytometer and Cell Quest software.

### TUNEL Assay

TdT-UTP nick end labeling (TUNEL) assays were performed with the one-step TUNEL kit (Roche, Switzerland), according to the manufacturer’s instructions. Transfected HepG2 cells were fixed onto poly-(L-lysine)-coated slides with 4% paraformaldehyde. Slides were rinsed with PBS, and cells were then permeabilized with 0.1% Triton X-100. Slides were washed with PBS and cells were incubated in 50 µl of TUNEL reaction mixture for one hour at 37°C in the dark. Next, 50 µl of DAPI was added and incubated for two minutes at room temperature. Cells were imaged by fluorescent microscopy using 488 nm excitation and 530 nm emission. Cells exhibiting green fluorescence were defined as TUNEL positive, apoptotic cells.

### Cell Invasion and Migration Assays

The invasive potential of HepG2 cells was evaluated using transwell inserts with 8 µm pores (Coring, NY, USA). Briefly, transfected cells were re-suspended in serum-free DMEM medium at a density of 1.0×10^6^/ml. 300 µl Cell suspension and 500 µl DMEM containing 20% FBS were respectively added to each insert and the matched lower chamber. After 48 hours, non-invading cells were removed using a cotton swab, and then the underside of the insert was stained. Six random fields for each insert were counted. For the migration assay, the procedures were similar to those of the invasion assay, except that 200 µl cell suspension was cultured in each transwell insert without matrix gel pre-coated.

### Statistical Analysis

SPSS version 13.0 for Windows was used for all analyses. The Chi-squared test was used to examine possible correlations between TRIM24 expression and clinicopathological factors. The Student’s t-test was used to compare other data. p value was based on the two-sided statistical analysis, and p<0.05 was considered to indicate statistical significance.
